# Mixed emotions: binary paths of humble leadership influencing employee behavior

**DOI:** 10.3389/fpsyg.2024.1431713

**Published:** 2025-01-24

**Authors:** Yanping Shen, Wenbing Wu, Shufei Xu, Yi Wang, Fangyuan Cai

**Affiliations:** ^1^School of Electronic and Information Engineering, Beijing Jiaotong University, Beijing, China; ^2^School of Economics and Management, Beijing Jiaotong University, Beijing, China; ^3^School of Journalism and Communication, Huaqiao University, Xiamen, China; ^4^China Galaxy Securities Co., Ltd., Beijing, China

**Keywords:** humble leadership, Social Information Processing Theory, time theft, innovative behavior, supervisor's organizational embodiment

## Abstract

The importance of humble leadership has garnered increasing attention among researchers. Most existing research focuses on its positive effects, but its negative effects are rarely discussed. From a more dialectical perspective, this study found that humble leadership has a dual impact, as it can foster employee innovative behavior and also trigger instances of time theft. Based on social information processing theory, this study used a questionnaire survey and structural equation modeling. Through the data analysis of 303 sample, the results suggest that humble leadership can reduce employee time theft by reducing employees' perceived acceptance of norm violations, while stimulating employee respect can increase employee innovative behavior, as well as supervisor's organizational embodiment can have a positive moderating effect. This study examines the mechanism of the influence of humble leadership on employee innovative behavior and time theft, broadens the body of humble leadership research, reveals the role of supervisor's organizational embodiment, and provides theoretical insights for enterprises to optimize organizational management.

## 1 Introduction

In today's fiercely competitive business landscape, the imperative to eradicate negative behaviors within organizations and inspire employees to actively engage in their work presents a formidable challenge for many companies. Effective leaders typically exhibit strong self-mastery and serve as exemplary role models, exerting a profound influence on the behavior of employees within the organization (Hannah et al., 2011). Humble leadership, as an effective leadership style, has gradually gained widespread attention from both academia and practice (Chandler et al., 2023). Humble leadership not only promotes employee innovation and enhances work engagement through the leader's knowledge-sharing behaviorse (Al Hawamdeh, 2024), but also proves more effective in mitigating negative behaviors such as time theft by fostering high-quality exchange relationships between leaders and subordinates. This is achieved by enhancing employees' psychological empowerment and reducing cynicism (Lorinkova and Perry, 2017). Specifically, humble leadership stimulates team members' innovative thinking and cooperative spirit by demonstrating humility and addressing employees' genuine needs, thereby improving overall work efficiency and creativity (Mrayyan and Al-Rjoub, 2024). Consequently, leaders must adjust their leadership styles to guide their teams in a more positive and constructive direction, thereby unleashing the full potential of their employees and collectively fostering organizational growth.

The essence of “bottom-up leadership” lies in humility (Collins, 2005; Matteson and Irving, 2006), wherein leaders cultivate an environment where employees feel respected, valued and appreciated. Humble leadership, a relatively novel concept in the realm of “bottom-up” leadership, has evolved into an independent leadership approach. It is characterized by a leader's capacity to objectively assess themselves, acknowledge and appreciate the strengths and contributions of others, and exemplify humility and respect. Furthermore, humble leaders lead by example, demonstrating teachability and serving as role models for their employees (Owens et al., 2013). They willingly devolute their authority and foster mutual growth with their teams (Owens and Hekman, 2012). Embracing new ideas, suggestions, and information, they exhibit a keen eagerness to learn from others (Tangney, 2000). This leadership style not only establishes a platform for equitable dialogue with employees but also positively evaluates and motivates their contributions and achievements, profoundly influencing the psychological well-being and behavior of employees (Hassan et al., 2023). Therefore, delving into how humble leadership shapes employee perceptions and behaviors through its distinctive attitudes and behavioral patterns is of paramount importance.

Previous research has predominantly highlighted the positive impact of humble leadership within organizations. It suggests that humble leaders can serve as motivational catalysts (Ma et al., 2020), fostering psychological empowerment among employees (Chen et al., 2018) and thereby fostering positive work behaviors (Kelemen et al., 2023). Moreover, humble leadership is linked to increased employee satisfaction, prioritization of employee career development (Owens and Hekman, 2012), and heightened commitment to their work (Tierney et al., 1999). Humble leaders exhibit a willingness to acknowledge their own shortcomings while valuing the strengths and contributions of their employees, thereby motivating employees to cultivate psychological capital (Ma et al., 2020) and experience greater autonomy and engagement (Owens and Hekman, 2012). These findings imply that humble leadership fosters feelings of respect and empowerment by acknowledging and recognizing employees' contributions and suggestions. Through their daily interactions and emphasis on employee growth and development, as well as by identifying and commending employees' strengths, humble leaders profoundly influence employees' psychological well-being and work performance. In essence, we attempt to offer a nuanced understanding of the relationship between humble leadership and employee behavior, contending that leaders' demonstration of humility traits inspires positive work behaviors among employees.

It is worth noting that while Mallen's research suggests that humble leadership yields positive outcomes (Mallén et al., 2019), other scholars have proposed that humility in leadership may also have negative consequences (Ali et al., 2021). Prior studies have underscored the importance of investigating whether humility in leadership leads to adverse effects (Ou et al., 2018; Weidman et al., 2018; Ali et al., 2021), particularly concerning employees' work time management behaviors. Some researchers express optimism, asserting that leader humility significantly correlates with reduced employee procrastination (He et al., 2023). However, others adopt a more cautious stance, positing that adequate resources and support motivates employees to work efficiently and minimize delays (Metin et al., 2019). They suggest that leadership humility may foster employee's psychological empowerment but could also exacerbate deviant behaviors in the workplace (Qin et al., 2020). Notably, time theft is recognized as a form of negative deviant behavior, yet many employees still engage in personal tasks during work hours (Martin et al., 2010). In addition, perceptions of resource importance vary across different work environments (Halbesleben et al., 2014). In new media organizations, employees often rely on internet browsing to obtain resources for productions, potentially increasing the likelihood of internet abuse (Lim, 2002) and workplace distraction. Research indicates that office employees spend over 1.3 h per day on personal activities or internet surfing, resulting in substantial productivity losses of ~$8,875 per year for employees (d'Abate and Eddy, 2007). Considering these findings, this paper contends that leader humility can influence employees' perceptions and negatively impact work time management behaviors.

The Social Information Processing (SIP) Theory (Salancik and Pfeffer, 1978; Zalesny and Ford, 1990) provides a comprehensive framework for understanding these dynamics. The theory emphasizes individuals' adaptability, suggesting that they can adjust their behaviors and attitudes in response to various factors such as the external environment and personal experiences (Frazier and Bowler, 2015; Lau and Liden, 2008). It posits that people engage in intricate cognitive and mental processes when interpreting social information, which fundamentally shapes their subsequent behaviors and attitudes.

Within an organization, employees are initially influenced by instructions and statements from their superiors, while concurrently being influenced by the prevailing organizational culture, including its norms, values and goals. Essentially, leaders' attitudes and behaviors attract employees' attention, which they then interpret and internalize, often imbuing them with emotional significance and thereafter mold their own behaviors. In this context, social support information plays a pivotal role in shaping individual's emotional responses and decision-making processes. According to the Social Information Processing Theory, social information is not simply transmitted; rather, it undergoes cognitive and affective processing, which profoundly impacts employees' emotional commitment, behavior, and attitudes. Consequently, addressing negative employee behaviors and fostering positive engagement emerges as a significant challenge for organizational management (Kang et al., 2023).

This study aims to leverage the SIP theory to elucidate when and why leader humility elicits varied employee attitudes and subsequent behaviors. Specifically, it posits that humble leadership fosters feelings of respect among employees, thereby encouraging their active participation in organizational development, ultimately fostering innovative behavior. However, it also acknowledges that humble leadership, by recognizing limitations and praising employees' strengths, might inadvertently inflate employees' self-competence and self-worth. This inflation could lead to a heightened sense of entitlement (Campbell et al., 2004; Vincent and Kouchaki, 2016), potentially resulting in employees perceiving leaders as more tolerant of rule violations (Qin et al., 2020), thus inducing increased incidents of time theft.

This study makes four significant contributions: First, it adopts the Social Information Processing Theory as a theoretical framework and constructs an integrated model depicting the dual pathways through which humble leadership influences employee behavior. By exploring the nuanced role of humble leadership as both a catalyst for promoting innovative behavior and a potential driver of increased time theft, this study offers a more comprehensive and balanced understanding, deepening our insights into the mechanisms underlying the impact of humble leadership. Second, it elucidates that employees' perceived acceptance of norm violations by leaders as a positive mediator between humble leadership and employee time theft, while perceived respect acts as a positive mediator between humble leadership and employee innovative behavior. These findings provide crucial insights into the multifaceted nature of humble leadership's impact in the workplace. Third, the study examines the moderating role of leadership style in shaping employees' perceptions of norm violations acceptance and the positive influence of perceived respect. This empirical evidence offers theoretical support and practical guidance for organizations seeking to implement humble leadership practices effectively. Fourth, the research uncovers distinct responses of employee innovative behavior and time theft to leadership styles within the context of Chinese new media organizations. These findings provide valuable managerial insights for optimizing institutional management strategies within the rapidly evolving landscape of new media organizations.

## 2 Literature review and hypothesis development

### 2.1 Social Information Processing Theory and humble leadership

Social Information Processing (SIP) Theory was first proposed in 1978. It posits that social information is derived from observing others' behavior, listening to their perspectives, and gathering information about the task environment as well as the effects of getting along with others (Salancik and Pfeffer, 1978). It underscores the role of organizational context in shaping individuals' goals, rules, and attitudes (Zalesny and Ford, 1990), highlighting the influence of the work environment on various employee responses and outcomes (Qiongjing Hu, 2018). Central to SIP is the notion that individuals actively or passively gather organizational information and process it for memory storage and cognitive judgments, thereby adjusting their attitudes and behaviors accordingly (Zalesny and Ford, 1990). Within this framework, humble leadership can be viewed as a significant source of organizational information, with leaders occupying a prominent position in shaping employee behaviors and attitudes (Yaffe and Kark, 2011). The behavioral and intrinsic traits exhibited by humble leaders create direct interpersonal interactions and convey organizational signals to employees. Thus, humble leadership can be conceptualized as a form of organizational information, wherein employees receive cues through interactions and subsequently adjust their behaviors and attitudes accordingly. This implies that humble leadership may exert both positive and negative impacts on employee behaviors, depending on the nature of these interactions. Individuals engage in three processes to process information from the external environment: learning, attribution, and judgment (Zalesny and Ford, 1990). Learning entails receiving and processing external information, which is then internalized as knowledge for storage and retrieval and influences subsequent subjective consciousness and objective behaviors (Bandura, 1977). Social information processing theory underscores the dynamic nature of this process: Firstly, diverse sources of information inherent in organizational culture, such as values, belief systems, codes of conduct, and strategic objectives, profoundly shape individuals' cognitive frameworks and meaning construction, prompting them to adapt their behaviors to align with organizational demands. Secondly, the interplay between different leadership styles and hierarchical relationships within the organization further influences employee attitudes and behaviors. Different leadership traits and behaviors establish different norms within the organizational environment, promoting employees to adapt their behaviors accordingly (Zalesny and Ford, 1990). Within the framework of Social Information Processing Theory, a leader's humble traits are viewed as a significant source of organizational information, transmitting organizational signals through direct interactions and shaping employee behaviors and attitudes (Rigolizzo et al., 2022). Additionally, humble leadership enhances follower rapport and fosters trust, thereby reducing negative behaviors such as knowledge hiding (Zhong et al., 2023). Humble leadership can also increase employees' work engagement by shaping their sense of efficacy and responsibility (Cheng et al., 2023). When leaders openly commend employees' achievements or encourage innovative initiatives, employees may develop an inflated sense of self-efficacy, leading to increased psychological empowerment (Harvey and Martinko, 2009). This perception may erroneously justify rule violations, as employees may believe that leaders exhibit tolerance toward such breaches. Consequently, employees may prioritize personal matters over formal work hours. Conversely, when leaders demonstrate genuine concern for employees' career development and advance with them, employees may perceive respect and appreciation, thus fostering a stronger sense of organizational belonging. In this case, employees are more likely to actively contribute to organizational growth and exhibit innovative behaviors that benefit the organization.

### 2.2 Perceived acceptance of norm violations and time theft

Norm violations encompass both physical and symbolic transgressions, spanning from breaches of social conventions to moral principles. Within organizations, employees' behavioral norms can become formalized organizational conduct rules through institutionalization (Morris et al., 2015; Yam et al., 2014). In the workplace, leaders wield considerable influence over employees' perceptions of normativity given their elevated status and formal authority, shaping employees' views on rule violations (Yam et al., 2018) and influencing their propensity to engage in deviant behaviors. When humble leaders genuinely acknowledge and commend the strengths and contributions of team members, their actions, while motivating, may inadvertently convey a cognitive bias. Employees may misinterpret the leader's emphasis on their value as an indication of high tolerance for minor transgressions, potentially leading them to engage in deviant behaviors such as time theft (Xu et al., 2023). The perception that supervisors are lenient toward rule violations may encourage employees to engage in such behaviors, anticipating minimal repercussions for their actions (Yam et al., 2018). Therefore, this study proposes the following hypothesis:

***H1:*** Humble leadership positively influences employees' perceived acceptance of norm violations by leaders.

Universal standards of professional ethics mandate that employees should demonstrate love for their jobs and maintain a high level of organizational engagement. However, the prevalent phenomenon of employees diverting their work time for personal activities persists (Martin et al., 2010; Gruys and Sackett, 2003), a behavior commonly referred to as time theft (Henle et al., 2010; Liu and Berry, 2013; Zhao et al., 2022a). Time Theft entails employees engaging in counterproductive activities or conducting personal affairs during their designated working hours, including intentional misrepresentation of their working hours while still receiving compensation from the company (Harold et al., 2022). Scholars estimate that employees waste 1–2 h per day on non-work-related activities (Henle et al., 2010; Martin et al., 2010). Given the intangible nature of time, managers often struggle to detect instances of time theft. This unethical behavior, motivated by self-interest, laziness, and skewed notions of fairness (Ketchen et al., 2008), poses significant financial burdens on organizations, with estimates suggesting that time theft costs U.S. companies over $100 billion annually (McGee and Fillon, 1995).

Despite its prevalence and substantial economic impact, the academic community has yet to fully investigate the specific manifestations and underlying factors driving time theft. Existing studies have predominantly explored the motives and causes of time theft from both organizational (Lorinkova and Perry, 2017) and employees' perspectives (Xu et al., 2023). From an organizational perspective, time theft is not only an ethical misconduct but also a serious breach of the internal rules and regulations (Harold et al., 2022; Liu and Berry, 2013). Strengthening penalties and enforcement mechanisms can effectively deter employees from engaging in negative work behaviors like time theft (Lawrence and Robinson, 2007). However, from an individual employee's standpoint, time theft may serve as a means to reclaim the leisure time taken up by work (Martin et al., 2010), motivated not solely by personal self-interest but also as a coping strategy to navigate demanding work environments (Xu et al., 2023).

Unfortunately, research has largely overlooked the impact of managerial perspectives on time theft behavior. In fact, the extent of time theft behaviors can vary significantly depending on leadership styles. In environments where leaders appear tolerant of minor rule violations, their attitudes and behaviors can subtly influence employees' perceptions of the organization's time management system. Employees may thus perceive certain deviations from the organizational rules as acceptable under specific circumstances, leading to increased incidences of time theft (Hu et al., 2023). This perception is reinforced when employees believe that such behavior is widely tolerated by their peers, leaders, and the organization (Buffalo and Rodgers, 1971). Drawing from social information processing theory, leader behavior serves as a key source of information for employees to evaluate the work environment. A leader's humble demeanor may foster a relaxed perception of the work atmosphere among employees. Perceiving the leader's accommodating attitude, employees may consciously align themselves with the leader's “in-group.” Consequently, employees may interpret certain behaviors of humble leadership as organizational acquiescence to time theft, viewing them as part of the organizational norms and culture. Driven by this perception, employees may be more inclined to engage in time theft, believing that they will face minimal repercussions even if detected. Based on this rationale, the following hypothesis is proposed:

***H2:*** Employees' perceived acceptance of norm violations by leaders mediates the relationship between the positive impact of humble leadership and employee time theft.

### 2.3 Perceived respect and innovative behavior

Respect entails positively recognizing and acknowledging the value of others, manifesting appreciation toward them, and acting accordingly (Downie and Telfer, 1969; Frankena, 1986). In society, respect holds deep-rooted significance and is universally cherished, with individuals aspiring to be respected both in personal and professional domains (Van Quaquebeke et al., 2009). Within organizations, respect addresses pivotal questions such as “how do I perceive my status within the organization” and “what is my social standing in the organization” (Tyler, 1999). Leaders, in this context, serve as key transmitters of social signals, with their words and actions profoundly shaping employees' perceived status within the company (Levinson, 1965; Grandey et al., 2002). Working under a leader who respects his or her team members constitutes one of the most cherished experiences in the work place, with “My work supervisor respects me” ranking the second most crucial work value (Van Quaquebeke et al., 2009).

Various facets of humble leadership contribute to employees' perception of respect. Firstly, humble leaders adopt a “bottom-up” communication approach, demonstrating humility by candidly acknowledging their limitations, admitting mistakes, and actively learning from them (Owens and Hekman, 2012). They genuinely appreciate and value the strengths and contributions of their team members, openly acknowledging and encouraging them. This egalitarian communication style naturally fosters a sense of respect among employees (Renger and Simon, 2011). Under the influence of strong leadership, perceived respect significantly promotes innovative behavior through thriving at work (Zhao et al., 2022b). Secondly, humble leaders prioritize creating an organizational environment where employees feel respected and have opportunities for growth. Employees' perceived growth prospects are closely intertwined with their experience of respect (Fuller et al., 2006), and humble leaders actively nurture and support their development (Vera and Rodriguez-Lopez, 2004). They closely engage with employees' career aspirations and collaborate with them, thereby augmenting employees' sense of respect within the organization. Thirdly, humble leaders demonstrate a receptive attitude toward new knowledge, ideas and feedback, promoting a culture of lifelong learning. They embrace and tolerate employees' innovative attempts and inevitable mistakes, fostering an environment conducive to innovation and personal values. This not only cultivates positive work sentiments but also amplifies employees' perceived respect for the organization and its leaders. Positive emotions stemming from respect enhance employees' perceptions of self-worth, motivating them to engage in innovative activities spontaneously, thereby contributing to overall organizational performance improvement. Self-worth is inherently tied to the experience of respect, and when employees possess a heightened sense of self-worth, they are more inclined to transcend their work roles' constraints and pursue personal value realization, thereby spawning more innovation (Lei, 2015). Conversely, inadequate respect from leaders, this unequal signaling (Van Quaquebeke et al., 2007), often triggers deviant behaviors (Bettencourt and Miller, 1996) and promotes self-interest over teamwork (Sleebos et al., 2007), engendering negative work attitudes and behaviors. Therefore, the interpersonal respect between leaders and employees is intricately linked to the collaborative and innovative atmosphere.

Aligned with social information processing theory, individuals evaluate their position in an organization based on their perceived information indicating whether they are “central, included, valued, and respected” (Tyler and Blader, 2002). When leaders are perceived as respectful, employees experience a sense of belonging, pride, and loyalty, fostering a conducive environment for innovation. Based on the above analysis, the following hypothesis is proposed:

***H3:*** Humble leadership positively influences employees' perceived respect.

In prior research, innovative behavior has been categorized into narrow and broad categories. Narrowly, it entails the generation of novel or creative thinking outputs by employees, while broadly, it encompasses the entire process from the initial innovative idea to its substantive implementation and grounding (Amabile et al., 1988; Amabile, 1996). This means that innovative behavior can either manifest in the generation stage of innovative thinking or extend to the complete realization of innovative ideas into tangible results. In recent years, leadership style has emerged as an important antecedent variable in understanding employee innovative behavior, with the interaction between leaders and employees significantly shaping this behavior (Han et al., 2023). Humble leaders, on the one hand, exhibit self-reflection and self-correction, dealing with their mistakes and limitations realistically. This not only fosters a culture of tolerance for mistakes but also encourages team members to embrace the unknown and actively explore new knowledge and technology (Kark and Carmeli, 2009). On the other hand, humble leaders appreciating employees' strengths, contributions and abilities can not only fulfill employees' psychological needs for self-worth but also greatly enhance their sense of respect, self-efficacy and organizational belonging (Nielsen and Huse, 2010). The leader's affirmation and respect serve as catalysts that inspire employees to maintain a positive and optimistic emotional state at work. This conducive emotional atmosphere encourages employees to explore their potential and contribute actively to the organization, thus fostering innovative behavior. Based on the above analysis, this study proposes the following hypothesis:

***H4:*** Employee perceived respect mediates the relationship between the positive influence of humble leadership and employee innovative behavior.

### 2.4 The moderating role of SOE incarnations

Supervisor's organizational embodiment (SOE) refers to the degree to which employees perceive their leader as the representation of the organization (Eisenberger et al., 2010), essentially acting as the organization's agent. In their daily work, leaders are tasked with communicating the organization's goals and mission to employees, overseeing adherence to relevant rules and policies, and achieving the organizational objectives. As a result, employees often interpret actions taken by organizational role models (representative leaders) and other leaders as “organizational behavior” (Levinson, 1965; March and Simon, 1958). Higher levels of SOE lead employees to perceive the way leaders treat them as reflective of the way the organization treats them. SOE, a perception held by individual employees, triggers different reactive behaviors based on how employees interpret social messages from leadership. In essence, SOE plays a moderating role in employees' processing of information sources in the work environment.

Previous research has demonstrated that SOE moderates the relationship between transformational leadership, a type of “bottom-up” leadership styles, and employee reactions (Eisenberger et al., 2010). Building on this, this study suggests that humble leadership, another “bottom-up” leadership style, can similarly moderate the interaction between leaders and employee reactions through SOE. When leaders show humility, employees may perceive such leaders as challenging the traditional image of organizational power, leading them to believe that moderate rule-breaking may not result in severe consequences. Likewise, when employees perceive high SOE from their leaders and receive positive interactions such as appreciation, attention, support, and motivation, they interpret these actions as organizational-level recognition of and respect for their individual contributions (Tyler, 1999). Specifically, they interpret leaders' acknowledgment of their contributions as organizational respect and validation of their personal values, thereby motivating them to higher levels of work engagement. Consequently, when employees with high SOE encounter humble leadership, they tend to directly link the leader's intentions and behaviors with the organization's overall objectives. Depending on how employees interpret this information, the leader's exemplary influence and authority will either be reinforced or diminished, consequently affecting employee's perceptual response. Recent studies have explored the role of SOE in the relationship between ethical leadership and organizational identification, finding that when employees perceive leaders as sharing organizational values and norms, leadership behaviors can effectively promote organizational identification and extra-role behaviors (Costa and Velez, 2022). Additionally, research has examined how ethical or abusive behaviors by upper-level managers influence SOE, revealing that ethical leadership promotes positive SOE, while abusive management leads to supervisors rejecting organizational values (Rice et al., 2023). Further studies have highlighted that SOE also moderates the relationship between humorous leadership and work engagement, thereby enhancing employee innovative behavior (Zhang and Su, 2020). Based on this, the following hypotheses are proposed:

***H5a:*** SOE moderates the positive relationship between humble leadership and employees' perceived acceptance of norm violations.

***H5b:*** SOE moderates the positive relationship between humble leadership and employees' perceived respect.

**Figure 1:**
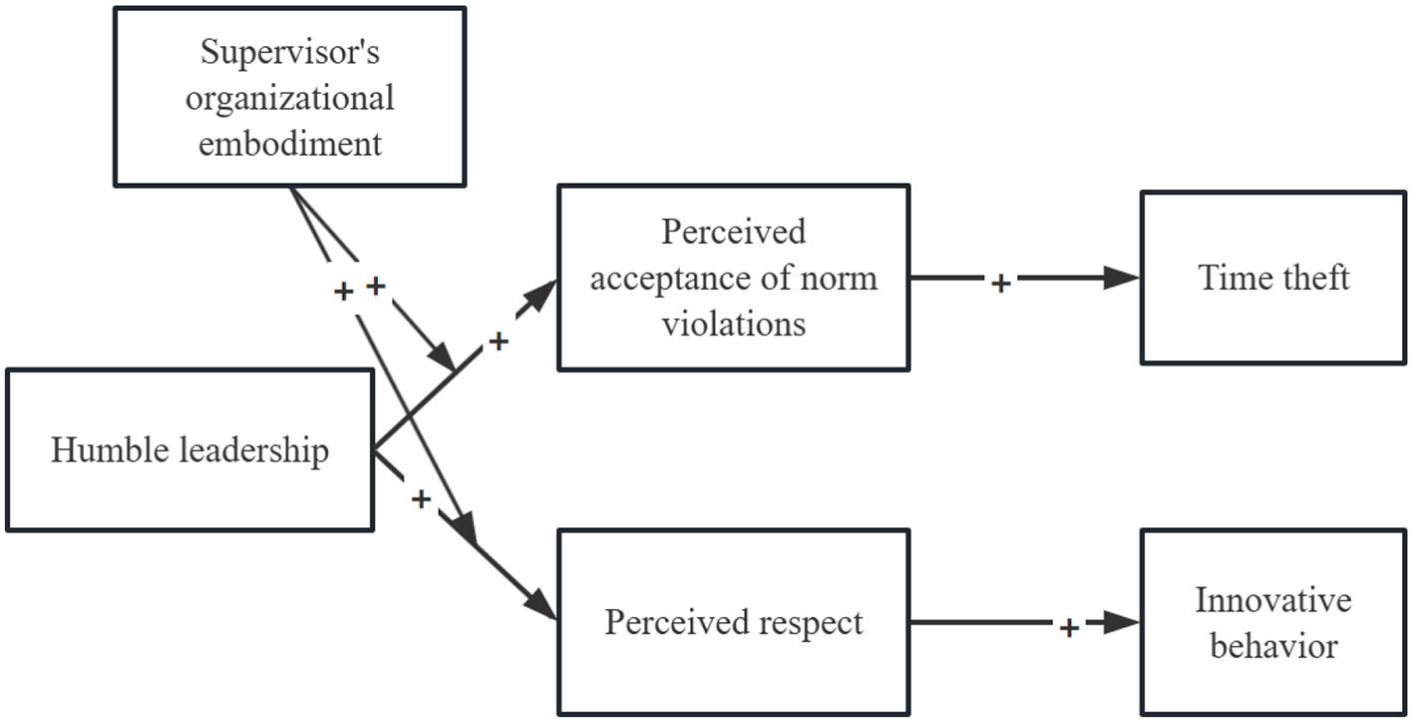
Theoretical model of this research.

In conjunction with the hypotheses, the effect of humble leadership on employee perceptions is moderated by the leader's organizational embodiment, resulting in different behavioral outcomes for employees. The higher the leader's humility, the more employees perceive the leader's tolerance of rule-breaking, potentially leading to increased time theft. At the same time, humble leadership that acknowledges others' achievements also promotes innovative behavior, as employees feel supported by the organization's strength and respected by their leader (see Figure 1).

## 3 Research methodology and research data

### 3.1 Variable measurement

To ensure the reliability and validity of the measurement instruments, established scales were used in this study. All scales were translated from English to Chinese strictly following Alberlin's (Brislin, 1970) back-translation procedure. A Likert 5-point scale was employed, ranging from 1 (strongly disagree) to 5 (strongly agree).

Humble leadership: we utilized a scale developed by Owens et al. (2013) (α= 0.94, kmo = 0.96) containing three dimensions: admitting one's own shortcomings and faults, appreciating the strengths and contributions of employees, and learning with humility. Each dimension comprised three items, resulting in a total of nine items. For example, “My leader recognizes that the knowledge and skills of others exceed his or her own.”

Perceived acceptance of norm violations: we employed a five-item scale (α= 0.77, kmo = 0.88) developed by Van Kleef et al. (2011). This scale gauges employees' perception of the leader's acceptance of behaviors such as being unsociable, unethical, inappropriate, rude, and impolite.

Time theft: a three-item scale (α= 0.87, kmo = 0.73) developed by Bennett and Robinson (2000) was utilized to measure employee time theft. Items such as “I spend too much time fantasizing or daydreaming at work” have been used by scholars in China (Zhao et al., 2022a).

Perceived respect: employees' perceived respect from their leaders was assessed using a scale developed by Van Quaquebeke and Eckloff (2010) (α= 0.96, kmo = 0.98). This scale consisted of 12 items, including statements like “My leader is polite to me” or “My leader values me and my work.”

Innovative behavior: we employed the employee innovative behavior measurement scale (α= 0.84, kmo = 0.92) revised by Zhang (2016) to suit the context of Chinese enterprises. This scale consisted of 8 items, such as “I often seek opportunities to improve my work methods and processes” and “I often introduce some new work methods to my colleagues.”

Supervisor's Organizational Embodiment (SOE): the scale developed by Eisenberger et al. (2010) (α= 0.90, kmo = 0.96) was used to measure the extent to which employees perceive leadership representation. This scale contained nine items, including “When a leader praises me, I feel that it is the company praising me” and “When a leader is satisfied with my work, I believe it is the company that is satisfied.”

Control variables: gender, age, educational background and years of experience in the field were chosen as control variables in this study. These variables are correlated with social support and information-seeking behavior to some extent and may influence the research results.

### 3.2 Samples and collection

This study adopted a combined approach of convenience and purposive sampling to select the sample. samples were collected through the WJX platform, a professional questionnaire survey platform in China. Based on the 44-item questionnaire, the sample size was determined to be between 300 and 350 responses, which is ~5–10 times the number of items in the survey. We selected the media industry for our survey due to the innovative, novel and uncertain nature of news products (Zhou, 2021), and because media practitioners enjoy greater autonomy over time and ideas in the production process. We identified the target group through online searches and reached out to some managers of media organizations. The study's purposes and significance were explained to them without disclosing any specific research hypotheses, and they were encouraged to participate actively. Additionally, we assured all employees that the study was for academic research only, that no private personal information would be disclosed, and that their responses would remain anonymous. In this way, we aim to establish a professional and rigorous research framework to thoroughly investigate the impact of humble leadership on specific employee behaviors. To mitigate common methodological bias in the data and to enhance the statistical validity of the causality tests, we collected data at three different time points, each ~2 weeks apart. At Time 1 (Nov. 7–Nov. 12, 2023), subjects completed a measure of leadership humility. At Time 2 (Nov. 28–Dec. 3), subjects were assessed for their acceptance of norm violations and perceived respect. At Time 3 (December 12–December 17), the questionnaire measured participants' engagement in time theft and innovative behavior at work.

A total of 350 questionnaires were distributed across the three surveys, and 313 questionnaires were returned, yielding a recovery rate of 89.4%. After excluding invalid questionnaires such as repeated answers, omissions and incomplete participation, we obtained 303 valid questionnaires, resulting in an effective recovery rate of 96.8%. The sample comprised 53.8% males and 46.2% females, with the majority of respondents under 40 years old (88.01%). Regarding education, the largest portion held undergraduate degrees (217 respondents, 71.62%), followed by master's degrees (63 respondents, 20.79%). In terms of work experience, the majority held <5 years of experience (93.07%), attributed partly to some media organizations being established since 2018, with many employees transferring from the traditional media industry. In terms of job level, ordinary employees account for the majority, with a total of 243 employees (80.19%), providing a solid sample background for the subsequent analysis, as detailed in Table 1.

**Table 1:** The demographic characteristics of samples.

**Sample characteristics**		***N***	**%**
Gender	Females	140	46.2
	Males	163	53.8
Age	25	36	11.88
	26–30	86	28.38
	31–35	102	33.66
	36–40	43	14.19
	40 and above	36	11.99
Education	College degree or below	23	7.59
	Undergraduate	217	71.62
	Graduate	63	20.79
Icome	Under $278	0	0
	$278–$417	0	0
	$417–$695	98	32.34
	$695 and over	205	67.66
Years of experience	1	31	10.23
	1–2	99	32.67
	2–3	75	24.75
	3–4	46	15.18
	4–5	31	10.23
	5 or above	21	6.93
Job level	Ordinary employees	243	80.19
	Group leader	50	16.5
	Department manager	10	3.3
	General manager	0	0

## 4 Results

This study employed structural equation modeling to assess the coherence of dependencies and to confirm causal links between multiple independent and dependent structures (Anderson and Gerbing, 1988). Data analysis was primarily conducted using SPSS 25.0 and Mplus 8.0. SPSS 25.0 is efficient for handling large-scale data and was used for reliability and validity testing, descriptive statistics, correlation analysis, factor analysis, and regression analysis. Mplus 8.0, with its significant advantages in handling complex models, multilevel data, and interaction effects, was employed for confirmatory factor analysis and moderation regression analysis, offering various estimation methods and robustness tests.

### 4.1 Preliminary analyses for Path 1

In Path 1, we examined the relationship between humble leadership, employees' perceived acceptance of norm violations by leaders, and time theft. The aim was to validate the distinct factor structure of each of the three core variables involved in Path 1, as shown in Table 2. The three-variable model comprising humble leadership, acceptance of rule violations, and time theft demonstrated good fit to the data (χ^2^ = 214.76, χ^2^/*df* = 1.85, *p* < 0.001, RMSEA = 0.05, CFI = 0.95). We conducted a two-by-two path analysis for two of the three variables: a two-factor model with humble leadership and perceived acceptance as a single factor (χ^2^ = 217.24, *p* < 0.001, RMSEA = 0.05, CFI = 0.95); and a two-factor model with perceived acceptance of norm violations and time theft as single factors (Δχ^2^ = 215.00, *p* < 0.001, RMSEA = 0.05, CFI = 0.95). In the one-way model, all three variables were set as single factors (Δχ^2^ = 218.38, *p* < 0.001, RMSEA = 0.05, CFI = 0.95). As depicted in Table 2, the fit of the three factors generally surpassed that of the other competing models, indicating excellent discriminant validity among the three variables examined in this study.

**Table 2:** Model fit for Path 1 (*n* = 303).

**Model**	**χ^2^**	**df**	**χ^2^/*df***	**CFI**	**TLI**	**RMSEA**	**SRMR**
Three-factor model (HL, PANV, TT)	214.76	116	1.85^***^	0.95	0.94	0.05	0.05
Two-factor model (HL + PANV, TT)	217.24	118	1.84^***^	0.95	0.95	0.05	0.05
Two-factor model (HL, PANV + TT)	215.00	118	1.83^***^	0.95	0.95	0.05	0.05
Two-factor model (HL + PANV + TT)	218.38	119	1.84^***^	0.95	0.95	0.05	0.05

^+^, two factors were combined; HL, humble leadership; PANV, perceived acceptance of norm violations; TT, time theft.

***p < 0.001.

Furthermore, we assessed the average variance extracted (AVE) and composite reliability (CR) of each dimension. As shown in Table 3, the standardized factor loading values of all measures on their corresponding dimensions fell within reasonable intervals. Meanwhile, the AVE and CR values of each of the three variables exceeded the critical thresholds of 0.7 and 0.5, indicating good convergent validity for all three variables.

**Table 3:** Confirmatory factor analysis results of Path 1 (*n* = 303).

**Constructs**		**Loadings**	**AVE**	**CR**
HL	HL1	0.83	0.63	0.94
	HL2	0.76		
	HL3	0.80		
	HL4	0.79		
	HL5	0.83		
	HL6	0.82		
	HL7	0.74		
	HL8	0.79		
	HL9	0.78		
PANV	PANV1	0.72	0.60	0.88
	PANV2	0.79		
	PANV3	0.75		
	PANV4	0.82		
	PANV5	0.79		
TT	TT1	0.78	0.64	0.84
	TT2	0.83		
	TT3	0.80		

HL, humble leadership; PANV, perceived acceptance of norm violations; TT, time theft.

We also conducted comprehensive descriptive statistics, reliability tests, and correlation analyses for each variable in Path 1. The reliability coefficients for humble leadership, perceived acceptance of norm violations, and time theft fell within the desirable range of 0.8 to 1, indicating excellent internal consistency of the scales used. Descriptive statistics revealed that the mean scores for acceptance of rule violations by humble leadership and employee engagement in time theft were moderate, with standard deviations indicating moderate variability.

These variables were scored on a positive scale of 1–5, suggesting that, on average, perceptions of humble leadership's acceptance of norm violations and employees' engagement in time theft were moderately high. For clarity and emphasis, we present bivariate correlations of these variables with the study variables in Table 4 to visualize the correlation between the two variables. Through a comprehensive examination of descriptive statistics, reliability tests, and correlation analyses, we gain a more accurate understanding of the nature and interrelationships of the variables in the model, providing robust support for subsequent hypothesis testing.

**Table 4:** Correlation for Path 1.

**Variables**	**Means (SD)**	**1**	**2**	**3**	**4**	**5**	**6**	**7**	**8**	**9**
1. Gender^a^	1.53 (0.50)	(-)								
2. Age	2.89 (1.18)	0.009	(–)							
3. Edu	2.02 (0.56)	0.16	0.19	(–)						
4. Wage income	3.69 (0.49)	–0.21^*^	0.18	–0.01	(–)					
5. Years of experience	3.49 (1.41)	0.01	.45^**^	0.21^*^	0.28^**^	(–)				
6. Job level	1.44 (0.63)	0.06	0.46^**^	0.27^**^	0.27^**^	0.48^**^	(–)			
7. HL	3.85 (0.93)	0.12	0.03	0.14	–0.09	0.04	0.08	0.94		
8. PANV	3.72 (0.87)	0.11	0.04	0.11	–0.14	0.10	0.08	0.93^**^	0.88	
9. TT	3.26 (0.62)	0.13	0.00	0.14	–0.10	0.10	0.12	0.94^**^	0.89^**^	0.84

^*^p < 0.05.

^**^p < 0.01.

^a^1 = Female, 2 = Male.

α are presented on the diagonal.

### 4.2 Tests of hypotheses for Path 1

Hierarchical regression analysis was conducted using SPSS 25.0 to examine the data for this research, and the regression results are presented in Table 5. In Model 1, time theft (TT) was employed as the dependent variable, while gender, age, educational background, wage income, years of experience and job level were included as control variables. The analysis revealed a significant positive effect of humble leadership (HL) on employees' engagement in time theft (β = 0.41, *p* < 0.001), confirming the validity of *H1*.

**Table 5:** Hierarchical regression results for Path 1 (*n* = 303).

**Variables**	**PNVA**		**TT**			
	**M2**		**M1**		**M3**	
	β	**t**	β	**t**	β	**t**
Gender^a^	0.02	0.72	–0.01	–0.25	–0.02	–0.29
Age	–0.01	–0.48	–0.02	–0.33	–0.02	–0.30
Edu	–0.03	–1.40	0.12	2.22	0.13^*^	2.31
Wage income	–0.06	–2.49	–0.01	–0.10	0.003	0.06
Years of experience	0.08^**^	2.95	–0.12^*^	–1.89	–0.14	–2.05
Job level	0.004^**^	0.16	–0.10^*^	–1.72	–0.10^*^	–1.73
HL	0.89^***^	33.92	0.41^***^	6.72	0.28^*^	2.06
PNVA					0.15^*^	1.09
*R* ^2^	0.85		0.15		0.15	
*F*	232.03		7.32		6.56	

HL, humble leadership; PANV, perceived acceptance of norm violations; TT, time theft.

^*^p < 0.05.

^**^p < 0.01.

^***^p < 0.001.

^a^1 = Female, 2 = Male

Moving to Model 2, while controlling for demographic variables, perceived acceptance of norm violations (PANV) by leaders was included as the dependent variable. Notably, HL exhibited a significant influence on PANV (β = 0.89, *p* < 0.001). Transitioning to Model 3, which built upon on Model 1 by incorporating PANV as an independent variable, both HL and PANV continued to exert positive effects (β = 2.06, *p* < 0.05; β = 1.09, *p* < 0.05, respectively), with the magnitude of their influence increasing. This suggests that the perceived acceptance of rule violations by leadership mediates the relationship between humble leadership and employee time theft, thus confirming the validity of both *H1* and *H2*.

To further probe the mediating role of perceived acceptance of norm violations by leadership, Model 4 in the PROCESS 3.5 program was employed to test its mediation in the relationship between HL and TT. As indicated by the analytical results in Table 6, the mediating role of PANV between HL and TT was confirmed through the Bootstrap technique, with an indirect effect value of 0.09 and a 95% confidence interval of (0.25, 0.40), which did not encompass 0, signifying the establishment of indirect effect. Additionally, the effect share analysis in Table 6 reveals that the PANV accounted for 23% of the effect, while the direct effect comprised 77%, thereby verifying *H2*.

**Table 6:** Mediation effect test for Path 1 (*n* = 303).

**Parameter**	**Estimate**	**SE**	**BootLLCI**	**BootULCI**	**Percentage**
Indirect effect	0.09	0.16	0.25	0.40	23%
Direct effect	0.30	0.16	0.02	0.62	77%
Total effect	0.39	0.07	0.26	0.52	

### 4.3 Discussion for Path 1

In our investigation of Path 1, we opted to examine leaders and employees within the new media industry as our research samples. Through a questionnaire survey, we delved into the extent of support for *H1* within domestic new media organizations amidst the backdrop of media convergence and China's unique social context. While this study holds practical significance, it's essential to acknowledge its limitations. Specifically, our focus was restricted to exploring the direct link between humble leadership and deviant behaviors, such as time theft, in the workplace, thus not encompassing all potential dimensions of influence.

Moving forward, we propose that humility may exhibit a “mixed blessing” effect. This implies that while humble leadership could be positively correlated with employee time theft, it may also spur positive work outcomes, such as enhancing employees' perceived respect and thereby fostering innovative work behaviors. To gain a more comprehensive understanding of this phenomenon, we suggest investigating how leaders' humility influences various aspects within Chinese new media organizations. Moreover, to further explore the potentially divergent impacts of humble leadership, we advocate for validating the second path of influence.

### 4.4 Preliminary analyses for Path 2

In Path 2, we aim to explore the relationship between humble leadership, employees' perceived respect, and innovative behavior at work. Before proceeding with hypothesis testing for Path 2, we conducted a series of confirmatory factor analyses (CFA) via Mplus for each variable in Path 2 to ensure that the unique factor structure among the three variables outperformed alternative models, as shown in Table 7. The three-variable model comprising humble leadership, employees' perceived respect and innovative behavior demonstrated a good fit to the data (χ^2^ = 441.72, χ^2^/*df* = 1.38, *p* < 0.001, RMSEA = 0.04, CFI = 0.98).

**Table 7:** Model fit for Path 2.

**Model**	**χ^2^**	**df**	**χ^2^/*df***	**CFI**	**TLI**	**RMSEA**	**SRMR**
Three-factor model (HL, PR, IB)	441.72	321	1.38^***^	0.98	0.97	0.04	0.04
Two-factor model (HL + PR, IB)	447.84	323	1.39^***^	0.98	0.97	0.04	0.04
Two-factor model (HL, PR + IB)	447.35	324	1.38^***^	0.98	0.97	0.04	0.04
Two-factor model (HL + PR + IB)	449.61	323	1.39^***^	0.97	0.97	0.04	0.04

^+^, two factors were combined; HL, humble leadership; PR, perceived respect; IB, innovative behavior.

^***^p < 0.001.

Specifically, a two-by-two path analysis was conducted on pairs of variables, resulting in a favorable fit for both the humble leadership and employees' perceived respect factors (χ^2^ = 447.84, *p* < 0.001, RMSEA = 0.04, CFI = 0.98), as well as for the employees' perceived respect and innovative behavior factors (Δχ^2^ = 447.35, *p* < 0.001, RMSEA = 0.04, CFI = 0.98). Similarly, the one-way model, where each variable was treated as a single factor, also exhibited a superior fit (Δχ^2^ = 449.61, *p* < 0.001, RMSEA = 0.04, CFI = 0.97), as presented in Table 7. These results indicate that the three variables in Path 2 possess good discriminant validity.

Similarly to Path 1, we further examined the average variance extracted (AVE) and composite reliability (CR) for each dimension. As shown in Table 8, the standardized factor loading indexes of the measurement items for each variable in Path 2 fell within acceptable ranges, and the AVE and CR values for all variables surpassed the critical thresholds of 0.7 and 0.5, respectively, indicating good convergent validity.

**Table 8:** Confirmatory factor analysis results of Path 2 (*n* = 303).

**Constructs**		**Loadings**	**AVE**	**CR**
HL	HL1	0.83	0.63	0.94
	HL2	0.76		
	HL3	0.80		
	HL4	0.79		
	HL5	0.83		
	HL6	0.82		
	HL7	0.74		
	HL8	0.79		
	HL9	0.78		
PR	PR1	0.83	0.64	0.95
	PR2	0.80		
	PR3	0.82		
	PR4	0.78		
	PR5	0.78		
	PR6	0.78		
	PR7	0.83		
	PR8	0.81		
	PR9	0.77		
	PR10	0.79		
	PR11	0.82		
	PR12	0.76		
IB	IB1	0.82	0.62	0.91
	IB2	0.81		
	IB3	0.82		
	IB4	0.78		
	IB5	0.73		
	IB6	0.78		

HL, humble leadership; PR, perceived respect; IB, innovative behavior.

Moreover, comprehensive descriptive statistics, reliability tests, and correlation analyses were conducted for each variable in Path 2, mirroring the approach taken for Path 1. As depicted in Table 9, the reliability coefficients for humble leadership, employees' perceived respect, and innovative behavior at work were all within the desirable range of 0.8 to 1, indicating excellent internal consistency of the measurement and scales. In addition, descriptive statistics on the variables of humble leadership for employees' perceived respect and innovative behavior at work was conducted, revealing mean scores between 3 and 4 on a positively scored scale of 1–5, with moderate standard deviations. This indicates that, on average, humble leadership exhibited moderately high levels in both employees' perceived respect and innovative behaviors at work, suggesting that employees perceive higher levels of respect and engage in more innovative behaviors under humble leaders. To succinctly highlight key points, we provide the correlations between the study variables in Table 9, offering a visual representation of their interrelationships. Through thorough analysis via descriptive statistics, confidence tests and correlation analysis, we gain deeper insights into the significance and associations among the variables in the model, laying the groundwork for subsequent hypothesis testing.

**Table 9:** Correlation for Path 2.

**Variables**	**Means (SD)**	**1**	**2**	**3**	**4**	**5**	**6**	**7**	**8**	**9**
1. Gender^a^	1.53 (0.50)	(−)								
2. Age	2.89 (1.18)	0.11	(−)							
3. Education background	2.02 (0.56)	–0.04	0.08	(−)						
4. Wage income	3.69 (0.49)	–0.06	0.16^**^	0.05	(−)					
5. Years of experience	3.49 (1.41)	0.04	0.43^**^	0.11	0.23^**^	(−)				
6. Job level	1.44 (0.63)	0.04	0.37^**^	0.10	0.21^**^	0.36^**^	(−)			
7. Humble leadership	3.85 (0.93)	0.13^*^	0.35^**^	0.00	0.04	0.42^**^	0.24^**^	0.94		
8. Perceived respect	3.83 (0.93)	0.13^*^	0.35^**^	0.01	0.04	0.43^**^	0.25^**^	0.95^**^	0.95	
9. Innovative behavior	3.82 (0.95)	0.12^*^	0.34^**^	–0.01	0.04	0.41^**^	0.22^**^	0.93^**^	0.94^**^	0.91

^*^p < 0.05; ^**^p < 0.01

^a^1 = Female, 2 = Male.

α are presented on the diagonal.

### 4.5 Tests of hypotheses for Path 2

This study used SPSS 25.0 for Hierarchical regression analysis to examine the hypotheses, with the regression data results shown in Table 10. Initially, in Model 1, innovative behavior was the dependent variable, with gender, age, educational background, wage income, years of experience and job level included as control variables. It was found that humble leadership (HL) had a significant positive impact on employee innovative behavior (IB) (β = 0.92, *p* < 0.001), confirming *H2*.

**Table 10:** Hierarchical regression results of Path 2 (*n* = 303).

**Variables**	**PR**		**IB**			
	**M2**		**M1**		**M3**	
	β	*t*	β	**t**	β	*t*
Gender^a^	0	–0.01	–0.01	–0.43	–0.01	–0.48
Age	0.003	0.16	0.01	0.51	0.01	0.48
Edu	0.01	0.55	–0.01	–0.37	–0.01	–0.70
Wage income	–0.02	–0.85	–0.003	–0.14	0.01	0.26
Years of experience	0.03	1.55	0.02	0.68	–0.001	–0.03
Job level	0.02	0.90	–0.01	–0.39	–0.02	–0.90
HL	0.93^***^	45.35	0.92^***^	38.53	0.42^***^	7.03
PR					0.54^***^	8.86
*R* ^2^	0.91		0.87		0.90	
*F*	400.35		282.15		321.72	

HL, humble leadership; PR, perceived respect; IB, innovative behavior.

^*^p < 0.05.

^**^p < 0.01.

^***^p < 0.001.

^a^1 = Female, 2 = Male.

Subsequently, in Model 2, while controlling for demographic factors, perceived respect (PR) of employees was assessed as the dependent variable, revealing a significant association between HL and PR (β = 0.93, *p* < 0.001). Model 3, incorporating PR as an independent variable for regression analysis, indicated that both HL and PR positively influenced innovative behavior (β = 0.42, *p* < 0.001; β = 0.54, *p* < 0.001), thus supporting the mediation of perceived respect in the relationship between humble leadership and employee innovative behavior, validating *H3* and *H4*.

To further scrutinize the mediating role of perceived respect, this study examined the mediating role of PR in the process of HL influencing IB using Model 4 in the PROCESS 3.5 program. As shown in Table 11, the Bootstrap technique confirmed the mediating role of PR between HL and IB, with an indirect effect of 0.52, and a 95% confidence interval (0.41, 0.64) that excluded 0, affirming the validity of the indirect effect. Additionally, the effect share analysis revealed that PR accounted for 54% of the effect, while the direct effect share was 46%, thereby validating *H4*.

**Table 11:** Mediation effect test for Path 2 (*n* = 303).

**Parameter**	**Estimate**	**SE**	**BootLLCI**	**BootULCI**	**Percentage**
Indirect effect	0.52	0.06	0.41	0.64	54%
Direct effect	0.44	0.06	0.32	0.56	46%
Total effect	0.96	0.02	0.92	1	

**Figure 2:**
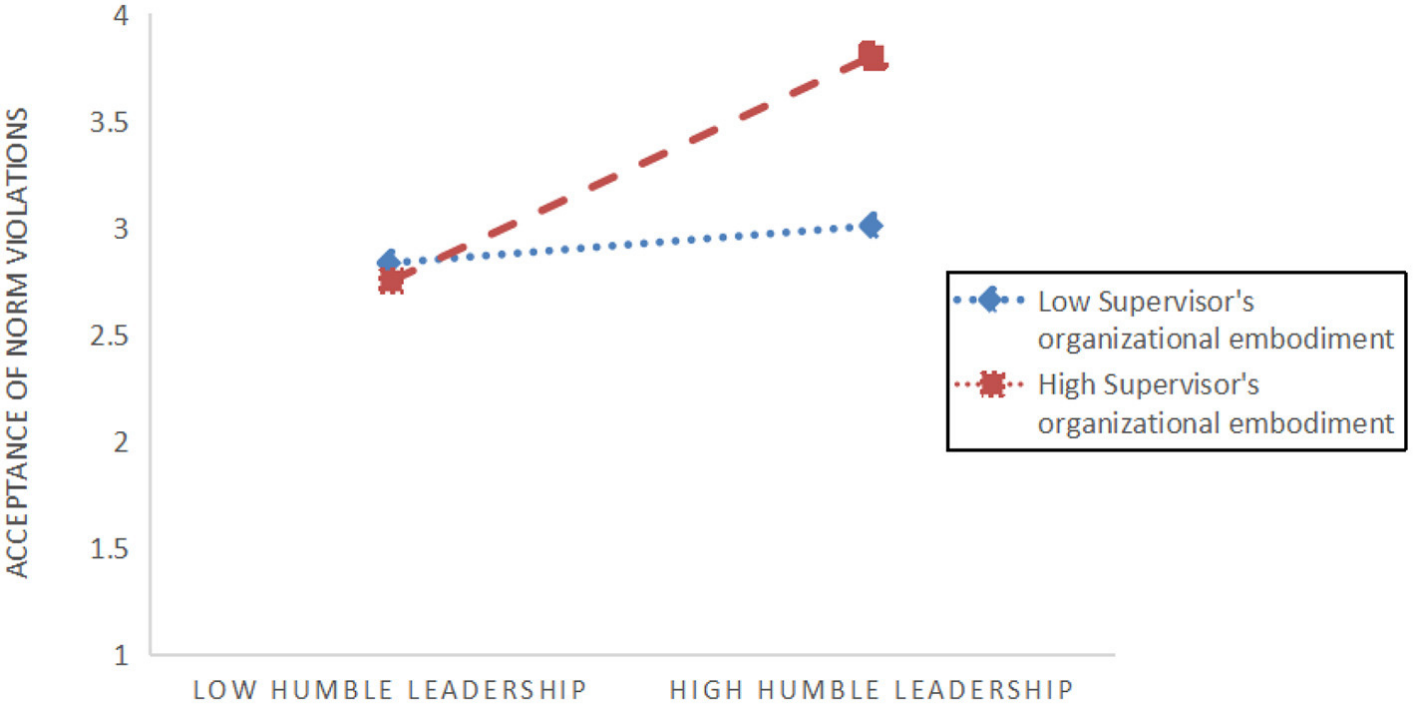
Decomposition diagram of regulatory effects for Path 1.

To examine *H5a*, we assessed the interaction between supervisor's organizational embodiment (SOE) and humble leadership on perceived acceptance of norm violations. In the first step, both humble leadership (β = 0.50, *p* < 0.001) and SOE (β = 0.44, *p* < 0.001) exhibited positive associations with perceived acceptance of norm violations (refer to Table 12). Subsequently, in the second step of regression, upon integrating interaction terms for humble leadership and SOE, the model demonstrated significantly greater explained variance (adjusted *R*^2^ = 0.87; Δ*R*^2^ = 0.01, *p* < 0.05) and a significant interaction term (β = –0.36, *p* < 0.001). For clarity, we visualized the interaction effect in Figure 2, confirming its anticipated direction. Further analysis was conducted following the approach outlined by Hayes (2017), stratified according to one standard deviation above and below the moderator mean. Notably, the mediating effect was significant when SOE was low (conditional indirect effect = 0.50, SE = 0.06, 95% CI = 0.37–0.62). Similarly, the mediator model remained significant when SOE was high (conditional indirect effect = 0.08, SE = 0.11, 95% CI = 0.14–0.30). Moreover, the index of moderate mediation was significant (index = 0.29, SE = 0.08, 95% CI = 0.14–1.05). These findings indicate that the influence of humble leadership on time theft behavior is heightened when the leader strongly represents the organization and attenuated when the representation is weak, thereby confirming *H5a*.

**Table 12:** Regression analysis of moderating effect for Path 1.

**DV = PNVA**				
**Variables**	**M1**		**M2**	
	β	*t*	β	*t*
HL	0.50^***^	7.23	0.31^***^	3.77
SOE	0.44^***^	6.24	0.18^***^	3.63
HL X SOE			0.22^***^	4.34
X SOE adjusted *R*^2^	0.86		0.87	
F	899.08^***^		641.35^***^	

HL, humble leadership; PANV, perceived acceptance of norm violations; SOE, supervisor's organizational embodiment.

^*^p < 0.05.

^**^p < 0.01.

^***^p < 0.001.

**Figure 3:**
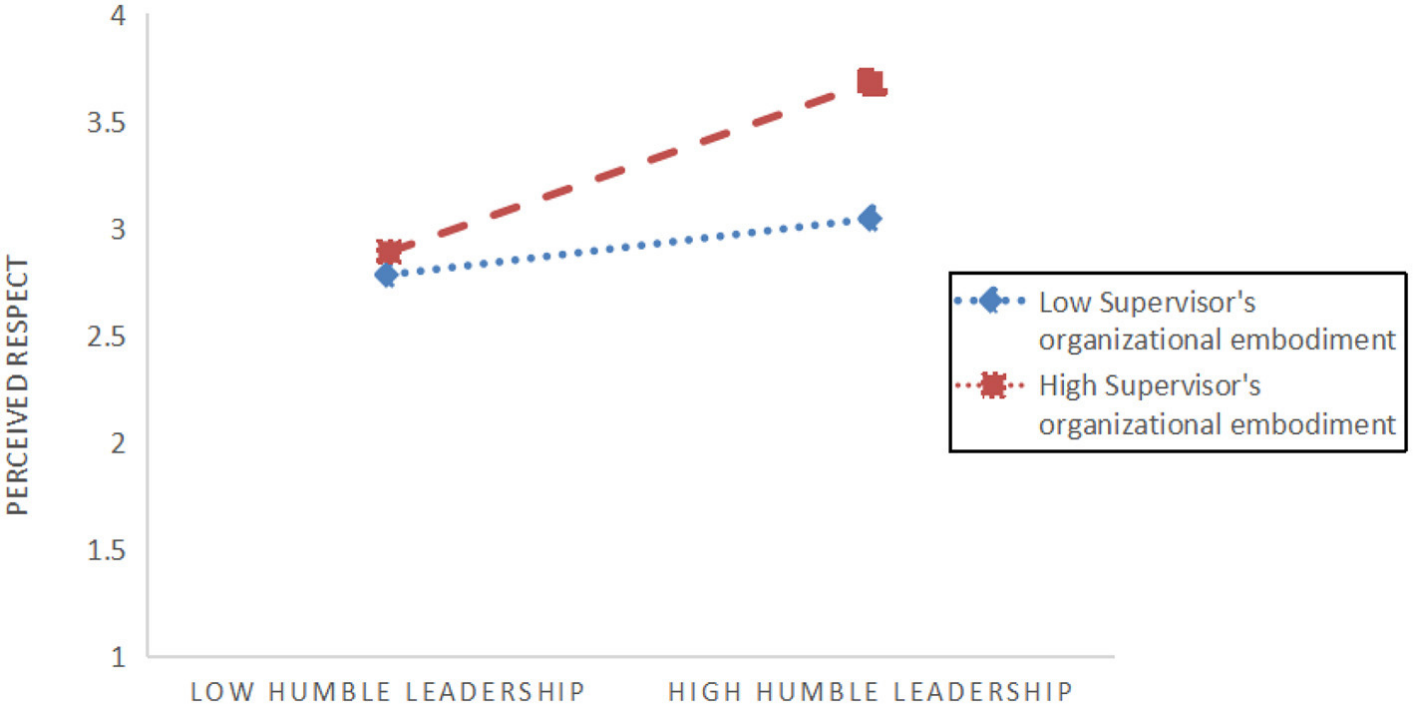
Decomposition diagram of regulatory effects for Path 2.

**Figure 4:**
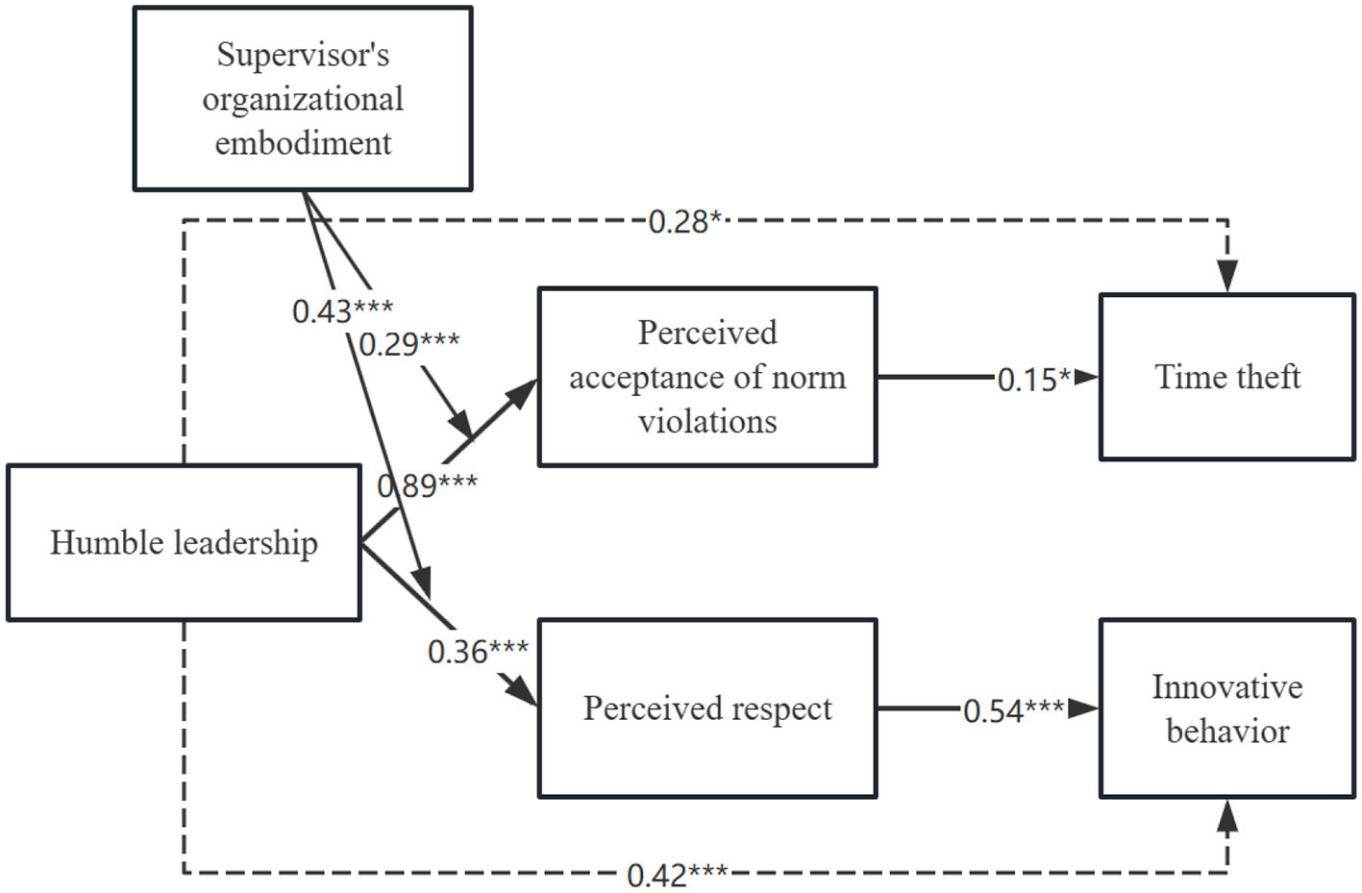
Results of path study. Unstandardized path estimates are reported. Solid lines depict the hypothesized relationships and dashed lines indicate relationships that are not hypothesized. ^*^p < 0.05, ^**^p < 0.01, ^***^p < 0.001.

To test *H5b*, the interaction between leadership SOE and humble leadership on employees' perceived respect was analyzed. In the first step, both humble leadership (β = 0.5, *p* < 0.001) and leadership organizational incarnation (β = 0.47, *p* < 0.001) demonstrated positive associations with employees' perceived respect (refer to Table 13). In the step 2 of regression, upon incorporating the interaction terms for humble leadership and SOE, the model exhibited significantly greater explained variance (adjusted *R*^2^ = 0.94; Δ*R*^2^ = 0.01, *p* < 0.05) and a significant interaction term(β = –0.43, *p* < 0.001). To facilitate interpretation, we visualized the interaction effects in Figures 3, 4. We then utilized the approach outlined by Hayes (2017), stratifying the analysis based on one standard deviation above and below the moderator mean. Notably, the mediating effect was significant when the leader's level of organizational incarnation was high (conditional indirect effect = 0.001, SE = 0.08, 95% CI = 0.44–0.62). Similarly, the mediator model remained significant when SOE was low (conditional indirect effect = 0.53, SE = 0.05, 95% CI = 0.06–0.16). The index of moderate mediation was also significant (index = 0.27, SE = 0.06, 95% CI = 0.16–0.38, refer to Table 14). These findings suggest that when humble leadership is associated with employees' perceived respect, the extent to which leaders represent the organization influences the level of respect perceived by employees, subsequently affecting innovative behavior. Thus, *H5b* is supported.

**Table 13:** Regression analysis of the moderating effect for Path 2.

**DV = PR**				
**Variables**	**M1**		**M2**	
	β	*t*	β	*t*
HL	0.50^***^	9.93	0.26^***^	4.76
SOE	0.47^***^	9.40	0.19^***^	5.48
HL X SOE			0.13^***^	7.64
X SOE adjusted R^2^	0.93		0.94	
*F*	1853.54^***^		1492.04^***^	

HL, humble leadership; PR, perceived respect; SOE, supervisor's organizational embodiment.

^*^p < 0.05.

^**^p < 0.01.

^***^p < 0.001.

**Table 14:** Overview of indirect effects and conditional indirect effects.

**Paths effects**	**Estimates**	**SE**	**95% confidence intervals**
**HL**→**PNVA**→**TT**			
Direct effect	0.29	0.08	(0.14, 1.05)
**Moderate adjustment**			
High SOE	0.08	0.11	(0.14, 0.30)
Low SOE	0.50	0.06	(0.37, 0.62)
**HL**→**PR**→**IB**			
Direct effect	0.27	0.06	(0.16, 0.38)
**Moderate adjustment**			
High SOE	0.001	0.08	(0.44, 0.62)
Low SOE	0.53	0.05	(0.06, 0.16)

## 5 Discussion

This study offers an in-depth analysis of the dual impact of humble leadership on employees' time theft and innovative behavior. Drawing on social information processing theory as the theoretical framework, it elucidates the mechanism through which humble leadership affects employees' mindset and behavior, both positively negatively. On the positive side, humble leadership effectively fosters the perceived respect by employees, thereby stimulating their innovative behavior.

Conversely, the humility exhibited by leaders may inadvertently signal a higher tolerance for minor rule-breaking, potentially inducing time theft behavior among employees. Additionally, SOE plays a positive moderating role in both perceived acceptance of norm violations by leaders and perceived respect. Specifically, this study empirically found that:

Firstly, humble leadership positively affects employees' perceived acceptance of norm violations by leaders. While humble leadership is commonly associated with positive leadership traits, such attitudes and behaviors may inadvertently signal to employees a leniency toward norm violations and deviant conduct. This unintended consequence could lead to blurred organizational standards, potentially undermining organizational effectiveness and discipline. Previous research has highlighted the role of inclusive leadership in fostering employees' psychological security (Khattak et al., 2022). Building on this, this study further explores how humble leadership, characterized by inclusive characteristics, positively influences employees' perceived acceptance of norm violations by their leaders. According to Social Information Processing Theory, leader's behaviors shape employees' psychological states. When employees perceive higher levels of psychological support (Javed et al., 2017), their inclination to engage in rule-violating behaviors may increase, ultimately leading to more deviant behaviors.

Secondly, employees' perceived acceptance of norm violations by leaders mediates the relationship between the positive impact of humble leadership and employee time theft. By demonstrating humility toward employees, leaders may inadvertently convey the impression that they are open to flexible enforcement of rules, owing to the employees' own strengths and contributions. By uncovering the underlying mechanism of how humble leadership affects employees' time management behaviors, we shed light on the potential for time theft among employees. This not only enriches our theoretical understanding of time theft in the workplace but also further broadens the application of humble leadership across various work contexts. Previous studies have predominantly investigated rule violation acceptance as either an independent or dependent variable (Khattak et al., 2022; Khan et al., 2023), with limited focus on its role as a mediating variable. Our study further substantiates the mediating role of perceived acceptance of norm violations by leaders in the relationship between humble leadership and employee time theft. When humble leaders acknowledge employees' accomplishments, employees may interpret it as a sign of their strengths being valued and mistakes being tolerated. Consequently, employees may perceive that engaging in deviant behaviors like time theft carries minimal repercussions, thus leading to an increase in time theft during working hours. For employees, engaging in time theft may be justified by the notion of “no theft, no gain”, leading them to reduce their work effort under the belief that the consequences of time theft are negligible (Martin et al., 2010).

Thirdly, humble leadership positively influences employees' perceived respect. Employees' deepest desire is to encounter a leader who truly respects them (Van Quaquebeke et al., 2009), understands their needs and values, as well as fosters an environment of dignity and respect. In such a setting, employees feel acknowledged and valued, thereby becoming more motivated to perform to their fullest potential. At the core of humble leadership, characterized by sincerity, openness and respect for others, lies the establishment of a sense of equality (Renger and Simon, 2011). Humble leaders do not merely view their employees as subordinates, but rather as partners to learn from and respect with an inclusive mindset. This approach significantly diminishes the psychological distance between superiors and subordinates, enabling employees to genuinely perceive respect and care from their leaders. From the viewpoint of social information processing theory, this represents a positive cognitive process where employees internalize leadership behaviors into their own feelings.

Fourthly, employees' perceived respect serves as a mediating factor in the relationship between the positive influence of humble leadership and employee innovative behavior. Through the lens of employees' perceived respect, this study explores how humble leadership influences employee innovative behavior. The analysis not only enriches theoretical insights into workplace innovation but also broadens the understanding of the relationship between humble leadership and innovative behavior. This study reveals how humble leadership influences employee innovative behavior, and underscores the important role of perceived respect in this process. When leaders treat employees with a high level of respect, it fosters not only emotional exchange but also cultivates a positive working atmosphere within the organization. By effectively positioning themselves, humble leaders appreciate and acknowledge the strengths and contributions of their employees (Owens and Hekman, 2012; Owens et al., 2013), providing positive feedback and encouragement. Such leadership behaviors significantly elevate employees' feelings of respect, trust, and belonging, instilling a profound sense of value and significance. Furthermore, when leaders consistently demonstrate a high level of respect for their employees, it fosters a deep impact on the employee to forge a strong connection with their identity (Sluss and Ashforth, 2008). This connection not only enhances employees' sense of identity and commitment to the organization but also fuels their desire to contribute more, thereby inspiring innovative behaviors. This leadership approach not only promotes personal growth and development among employees but also yields long-term benefits for the organization, such as enhancing the innovation and competitive advantage. In the environment of mutual respect, employees feel empowered to tackle workplace challenges with confidence and actively seek innovative solutions, thus driving organizational innovation and success.

Fifthly, SOE moderates the positive correlation between humble leadership and employees' perceived acceptance of norm violations. Prior research has primarily focused on exploring the moderating effects of SOE on leadership effectiveness in the contexts of leader-employee exchange (Eisenberger et al., 2014), transformational leadership (Eisenberger et al., 2010), and abusive management (Shoss et al., 2013). This study expands the interpretative scopes by introducing it to the realm of humble leadership for the first time. Previous studies predominantly relied on social exchange theory to explain the moderating role of SOE, positing that a high SOE amplifies the spillover effects of employees' social exchange and reciprocity. Consequently, when employees receive positive treatment from leaders, they respond based on the principles of reciprocity and social exchange, with avoidance of negative behaviors serving as a form of reciprocation (Shoss et al., 2013). This paper delves into a comprehensive investigation grounded in social information theory and unveils that the lenient attitude exhibited by humble leadership toward rule violations exerts a significant influence. When the level of SOE is high, humble leadership has the capacity to attenuate the rigid authoritative image of the organization to some extent, leading employees to perceive that “moderate breaches of rules are acceptable” in certain circumstances. This perception significantly influences employees' work attitudes and behaviors. Specifically, employees may interpret such cues as tacit approval or even encouragement of time theft in the workplace, prompting bolder and more uninhibited conduct. This phenomenon underscores the intricacies of rule enforcement and employee behavior management within the framework of humble leadership, highlighting the pivotal role of leaders in shaping organizational culture and employee behavior.

Sixthly, SOE moderates the positive correlation between humble leadership and employees' perceived respect. The essence of humble leadership lies in the intricate interplay between their behavior, organizational culture, and employee attitudes. Humble leaders epitomize caring, supportive, and respectful treatment for their employees; they are deeply committed to addressing employees' diverse needs and are also willing to empathize and grow alongside them. They provide employees with the necessary resources for their tasks and extend a helping hand in times of adversity (Brown and Treviño, 2006). Leaders with a high SOE are often viewed by employees as embodying the reputation and intangible status of the organization, conveying its fundamental beliefs and values. When high SOE leaders exhibit specific behaviors, employees tend to interpret them as organization-level signals reflecting the organization's expectations and attitudes. Consequently, when humble leaders demonstrate respect and empathy toward their employees, employees with a high in SOE perceive it not merely as the personal conduct of individual leaders, but also as indicative of the organization's overarching attitudes and values.

From the perspective of Social Information Processing Theory, this perception significantly influences employees' motivation and engagement. Having been respected and acknowledged at the organizational level, employees are more inclined to approach their work with a positive attitude and contribute to the organization's success and growth. They not only demonstrate dedication to the organization but also exhibit innovative thinking in their work, thereby fostering sustainable competitive advantage. Hence, SOE assumes a pivotal role in moderating the relationship between humble leadership and employees' perceived respect by nurturing a strong bond between employees and the organization, establishing a robust foundation for long-term organizational success.

## 6 Conclusions and implications

### 6.1 Conclusions

Humble leadership stands as a pivotal leadership style within organizational management, drawing extensive attention and scrutiny across various work contexts. Particularly within new media organizations, where employees wield considerable autonomy and decision-making authority in product production, the study of humble leadership's influence mechanism assumes paramount importance. Rooted in Social Information Processing Theory, this study delves deep into the relationship between humble leadership and employee behavior, gathering data and insights from new media organizations to scrutinize employees' perceptions of leadership's acceptance of norm violations and respect, and their impact on employees' time theft and innovative behavior. The following conclusions are drawn: (1) Humble leadership positively affects employees' perception of leaders' acceptance of norm violations. (2) Employees' perceived acceptance of norm violations by leaders mediates the positive influence of humble leadership on employee time theft. (3) Humble leadership positively impacts employees' perceived respect. (4) Employees' perceived respect mediates the positive influence of humble leadership on employee innovative behavior. (5) Leaders' SOE moderates the positive relationship between humble leadership and employees' perceived acceptance of norm violations by leaders. (6) Supervisor's organizational embodiment moderates the positive relationship between humble leadership and employees' perceived respect.

### 6.2 Management insights

First, judiciously exhibiting humility to leverage leadership's positive influence. Humble leaders, with their bottom-up leadership style, can foster employee respect, fuel creativity, and drive innovation. While they can mitigate deviant behaviors by exemplifying adherence to organizational norms, our study reveals both direct and indirect effects of humble leadership on employee time theft. Given humble leaders' inclination toward leniency regarding rule violations, they might inadvertently signal tolerance for such behavior, potentially prompting emulation by certain employees, thus fostering time theft. Therefore, leaders must temper their display of humble leadership and refrain from excessive rule-breaking. Although humble leadership may foster innovation, our findings underscore the need for leaders to remain vigilant against potential risks associated with humility. To clarify, leaders are not encouraged to abandon their humility in this study, but to exercise judiciously in avoiding excessive breaches of organizational rules. They should endeavor to foster a work environment that upholds norms and exhibits inclusivity while guiding employees toward adherence. By facilitating positive communication and guidance, leaders can underscore the significance of rules, enhance employees' rule awareness, underscore work importance and efficiency, thereby mitigating time theft. Businesses can train leaders on the potential negative impacts of excessive humility and encourage a more vigilant stance toward employees' work behaviors. Employees should be encouraged to be honest about work time and efficiency rather than engaging in time theft.

Second, respecting to employees for stimulating innovation. For leaders, demonstrating respect toward employees transcends professional decorum; it is the linchpin for nurturing team dynamics and enhancing organizational efficacy. Humble leadership significantly amplifies employees' perceived respect, thus catalyzing their contribution and creativity to the organization and fostering innovative behavior, and consequently driving personal and career development. When employees feel valued and acknowledged within the workplace, they are more willing to contribute novel ideas and solutions. This positive emotional feedback galvanizes proactive innovation and underscores sustainable organizational growth. While establishing work goals, leaders should proactively foster positive communication channels with employees, offer care and support to build a robust superior-subordinate relationship, thereby augmenting employees' innovative consciousness and capabilities (Han et al., 2023). Hence, the humble leadership style, albeit a double-edged sword, warrants consideration both as a moral imperative and a kind of management wisdom (Yanzi Wng, 2018). In daily management, leaders must remain attuned to the workplace and social cues emanating from their own words and behaviors, flexibly deploying this leadership style to amplify its positive effects and mitigates potential negatives. Leaders should reinforce organizational rules and socialization of employees to ensure rule adherence and effective implementation of innovative behaviors. A humble leadership style serves as a cost-effective strategy to foster a more inclusive and egalitarian workplace, outperforming structural management policies in motivating employees and promoting creativity, thereby driving sustainable organizational development. Consequently, within innovation-driven business management, promoting humble leadership can better stimulate the potential of employees, heighten organizational competitiveness, and spur organizational success.

Third, reinforcing leaders' alignment with the organization. In organizational settings, leaders transcend mere management roles; they embody organizational culture, safeguard values, and steer employee conduct. With their unique charisma, humble leaders emerge as the most influential and appealing figures within an organization. This underscores the imperative for humble leadership to not merely espouse organizational values verbally but also align actions with organizational ethos. Humble leadership prioritizes employee welfare, foster respect, and cultivates a positive work environment. When employees perceive humble leaders as organizational agents, they respect and trust them more, facilitating open sharing of ideas and suggestions. Perceiving leader's decisions and actions as reflective of organizational will and attitude can prompt employees to align with existing environmental information. A high tolerance for rule-breaking within the organization exacerbates employee propensity for deviant behaviors; conversely, when the organization espouses support and respect, employees endeavor to meet the expectations of the leader and the organization, showcasing heightened responsibility and initiative at work, and fostering innovation. Consequently, organizations should enhance employees' perceptions of leaders as organizational agents by augmenting support for leaders and fortifying managers' allegiance and identification with the organization, thereby maximizing the efficacy of humble leadership.

### 6.3 Limitations and perspectives

Grounded in the Social Information Processing Theory, this study reveals the processes underpinning employee time theft and innovative behavior at work, enriching our understanding of humble leadership's impact and leadership's organizational representation, and guiding companies in mitigating negative influences while stimulating employees' creativity. Nevertheless, this study harbors certain limitations. Within the binary path of humble leadership, other mediating factors may exist, warranting future exploration encompassing organizational climate, self-evaluation, role identity, work pressure, employee self-efficacy and other influence factors. Additionally, other influences can be scrutinized utilizing alternative theories such as Self-Determination Theory, Expectancy-Value Theory, and Attribution Theory. Furthermore, the extent to which humble leadership distinctly influences different employee perceptions and behaviors to reach the critical threshold warrants further investigation.

## Data Availability

The raw data supporting the conclusions of this article will be made available by the authors, without undue reservation.
